# Inventory management performance for family planning, maternal and child health medicines in public health facilities of West Wollega zone, Ethiopia

**DOI:** 10.1186/s40545-021-00304-z

**Published:** 2021-02-15

**Authors:** Oliyad Kebede, Gizachew Tilahun

**Affiliations:** 1 Department of Social Pharmacy and Pharmaceutics, School of Pharmacy, College of Medicine and Health Sciences, Mizan-Tepi University, Mizan-Aman, Ethiopia; 2 School of Pharmacy, Faculty of Health Sciences, Institute of Health, Jimma University, Jimma, Ethiopia

**Keywords:** Inventory management, Family planning, MCH medicines, Public health facilities

## Abstract

**Background:**

Inventory management is the heart of the supply system in improving availability of medicines, reducing the cost, and improving patient care quality. However, in the government facilities’ supply system, inventory management is poor. So, the purpose of this research is to assess inventory management performance for family planning, maternal and child health medicines in public health facilities of West Wollega zone, Oromia region, Ethiopia.

**Method:**

Facility-based descriptive cross-sectional quantitative study was conducted using checklist, structured and semi-structured questionnaire, and triangulated with qualitative method. Quantitative data were coded and analyzed using SPSS Version 20 and Microsoft excel spreadsheet. Qualitative data were analyzed manually, using thematic analysis technique. Different indicators were used to measure variables.

**Results:**

Among 23 health facilities assessed, availability of family planning/maternal and child health medicines ranged from 0 to 100%. Average availability of medicines was 14 (61.30%) with mean stock-out duration of 70.71 days. Bin cards were available for 559 (78.40%) of medicines, and 374 (52.45%) bin cards were accurate. Report submission rate was 116 (84.06%), with 47 (40.52%) report and resupply forms reported on time, 73 (62.93%) of them were complete and 69 (59.48%) were accurate. Supplier-related problem, lack of human resource, administrative problem, and lack of computer infrastructure were inventory management challenges identified.

**Conclusion:**

Inventory management performance for Family planning/maternal and child health medicines was poor as indicated by low availability, high stock-out duration, and poor LMIS performance. Efforts should be undertaken by concerned bodies to improve it.

## Background

Majority of the population in developing countries lack access to essential reproductive health medicines [1, 2]. In these countries, skilled health workers could not save mothers and children, due to chronic shortage of medicines. World Health Organization (WHO) report estimated that 8.1 million children under the age of five die every year and an estimated 1000 women die every day due to complications during pregnancy or childbirth in developing countries [3]. Ethiopia’s maternal mortality rate is among the highest in the world and 6.7 million women who want to avoid pregnancy do not use family planning [4].

Majority of these deaths are caused by postpartum hemorrhage (PPH), severe pre-eclampsia and Eclampsia, maternal and neonatal sepsis, sexually transmitted diseases (STIs), and unwanted pregnancy related complications, which could be averted with cost effective medicines [3–7]. To avail these medicines, inventory management plays a vital role. It is the heart of the supply system in improving availability of medicines, reducing cost, and improving patient care. However, the government facilities’ supply system inventory management is poor. Among inventory management challenges were: organizational and management structures, insufficient and poorly trained personnel, lack of information management, lack of robust management system, poor recording and reporting, and data not used for decision-making [8–13].

Therefore, by improving inventory management, it is possible to improve access to medicines. To improve inventory management performance, it is necessary to measure the current performance. This is done by considering the institutional context, describing desired performance, identifying gaps, root causes, and evaluating changes in performance [14]. But there is limited primary source of information regarding inventory management performance of public health facilities in Ethiopia.

So, the objective of this study was assessing inventory management performance at the public health facilities of the West Wollega zone. It assessed availability, logistics management information system, and challenges contributing to inventory management inefficiencies. There are very limited studies in Ethiopia and no study conducted in this typical study area regarding inventory management of FP/MCH medicines. It provides empirical snapshot for policy makers about current performance and track the future changes. It assessed FP/MCH medicines, unlike most of the previous studies that were emphasized on either FP alone or limited MCH commodities.

## Methods

### Study area and period

The study was conducted in selected public health facilities of West Wollega zone, Oromia regional state, west Ethiopia, from May 1, 2019 to May 30, 2019. West Wollega is located at western part of the country. It had 19 woredas with an area of 10,833.19 km^2^. The total population of West Wollega during the study period was estimated to be 1,350,415.

### Study design

Facility-based descriptive cross-sectional study was conducted using checklist, structured and semi-structured questionnaire and triangulated (supported) with qualitative study.

### Populations

The study populations were: selected public hospitals and health centers, selected FP/MCH medicines, all store managers in selected facilities, selected pharmacy heads, selected PHCU directors, selected hospital medical directors, selected supply chain coordinators, selected bin cards, and RRFs.

### Inclusion criteria

All hospitals and health centers that started service before May 1, 2018, all reporting and resupply forms (RRFs) and bin card used within a period of 1st May 2018 to 30th April 2019, and store managers of selected facilities were included for the quantitative study.

### Exclusion criteria

MCH medicines supplied under other programs were not included because they were supported by different partners, hence different in supply system.

### Sample size estimation

The sample size of the public health facilities was determined using USAID/DELIVER PROJECT guideline, logistics indicator assessment tool (LIAT). Accordingly, a minimum of 15% of the health facilities were included in the study [15]. There were five public hospitals and 68 HCs in West Wollega zone. Since hospitals are expected to serve more population and have more products all hospitals, that fulfill the inclusion criteria, were included. Regarding health centers, one health center from each woreda (19 health centers from 19 woredas) which is more than 15% (minimum sample size recommended by LIAT tool) was included. In general, 19 health centers and four hospitals, a total of 23 health facilities were assessed. Nine FP medicines from the latest Ethiopian Essential drug list and 22 MCH medicines from WHO priority list, a total of 31 FP/MCH medicines were assessed [2, 27]. The list of medicines included is available in Additional file 1.

### Sampling procedure

One health center was selected from each woreda using lottery method. For the in-depth interview, KIs who had a service of 2 years and above were purposively selected from hospital medical directors, PHCU directors, pharmacy heads, hospital supply chain coordinators, and store managers.

### Data collection tool

The data collection tools were adopted from Logistics System Assessment tool (LSAT) and Logistics Indicator Assessment Tool (LIAT) prepared by USAID/DELIVER Project [15, 16].

### Data collection procedures

Data were gathered through self-administrated questionnaires from store managers, filling checklists by observation and physical count of stocks, and review of relevant documents. Bin cards and RRFs utilized starting from May, 2018 to April 2019 were reviewed. The qualitative data were gathered through in-depth face-to-face interview with key informants. The interview guide was translated into the region’s working language, Afaan Oromo. Then, the interview was undertaken with Afaan Oromo and audio tape recorded. The result was translated into English by investigators. Each key informant was interviewed approximately for 15–20 min.

### Data management, analysis, and quality assurance

The quantitative data were checked for completeness of information and entered MS Excel 2016 spreadsheet and statistical package for social science (SPSS) version 20 to encode and analyze. The findings were summarized using tables and figures. The qualitative data were analyzed manually using the thematic analysis technique. Accordingly, the investigators familiarized with the recorded data by listening repeatedly and taking notes. Then we coded the data in Microsoft word table. The coded data were organized to search for subthemes. Finally, similar subthemes were brought together to form a theme. We described themes and quoted some KIs direct speech to strengthen our description. We trained data collectors for half a day and gave them strict instruction. We recruited Pharmacy professionals for data collection and supervised them.

### Operational definitions of terms

#### LMIS data quality

The extent to which the data recorded on the logistics recording and reporting tool is complete, accurate and reported on time [17].

#### Product availability

Products were considered available if product is not stock-out (if physical count is not zero) on the day of visit in the facilities’ store and dispensary.

#### Stock-out in previous 6 months

Medicines were considered stock-out if ending balance on logistics recording tool (bin card) is zero in previous 6 months from data collection time. For products with no bin or not updated the store managers were asked to recall if the medicines were stock-out and model 19 and 22 were reviewed.

#### LMIS forms utilization

LMIS forms were considered utilized if there were either manual or electronic forms on which the transactions of the FP/MCH products were recorded.

#### Timely reporting

Reports were considered timely if submitted within the first 10 days of the reporting month to PSA for hospitals and health centers that directly submit their report to PSA and within 5 days of PSA reporting month to woreda stores for HCs that submit their report to woreda stores.

#### Bin card accuracy

Bin cards were considered accurate if there was no discrepancy between stock recorded on bin cards and physical stock count and considered inaccurate if there was discrepancy.

#### RRF accuracy

RRFs were considered accurate if there were a mean discrepancy of 20% or less between balance on bin card and stock on hand recorded on RRF with corresponding period.

### Variables and their measurement

Variables in this study were measured by performance measurement indicators prepared by different organizations [26, 29, 30]. Details are available in Additional file 1.

## Results

### Socio-demographic characteristics

Twenty-three public health facilities (19 HCs and 4 hospitals) were surveyed to assess inventory management performance for FP/MCH medicines at West Wollega zone, Oromia regional state. Two of the hospitals were primary hospitals and the rest were general hospitals. In assessed health facilities, there were 77 health professionals with different professional backgrounds and educational levels under pharmacy units. From these professionals, 35 (45.4%) were druggists. The principal persons responsible for managing medicines were store managers. Majority (10 (43.5%)) of the store managers were nurses and (9 (39.1%)) of them had an experience of 1 to 5 years (Table 1).

**Table 1:** Socio-demographic characteristics of health professionals involved in logistics activities at Public health facilities of West Wollega zone, Oromia region, Ethiopia, May 2019

S. no.	Variables		Hospital frequency (%)	Health center frequency (%)	Total frequency (%)
1	Professionals under pharmacy unit	Pharmacist	24 (63.2%)	3 (7.7%)	27 (35.1%)
		Druggist	14 (36.8%)	21 (53.8%)	35 (45.4%)
		Nurse	0 (0%)	14 (35.9%)	14 (18.2%)
		Health Officer	0 (0%)	1 (2.6%)	1 (1.3%)
2	Service year of store managers	< 1 year	1 (25%)	4 (21.1%)	5 (21.7%)
		1–5 year	1 (25%)	8 (42.1%)	9 (39.1%)
		> 5 year	2 (50%)	7 (36.3%)	9 (39.1%)
3	Education qualification (staff of pharmacy unit)	Degree (B. Pharm)	24 (63.2%)	3 (7.7%)	27 (35.1%)
		Degree (note pharmacy)	0 (0%)	1 (2.6%)	1 (1.3%)
		Diploma (Pharmacy)	14 (36.8%)	21 (53.8%)	35 (45.4%)
		Diploma (Nurse)	0 (0%)	14 (35.9%)	14 (18.2%)
4	Store managers received training	IPLS	4 (100%)	14 (73.7%)	18 (78.3%)
		SC overview	2 (50%)	4 (21.1%)	6 (26.1%)
		Never trained	0 (0%)	5 (26.3%)	5 (21.7%)
5	Principal person managing medicines	Pharmacist	3 (75%)	1 (5.5%)	4 (17.4%)
		Druggist	1 (25%)	8 (42.1%)	9 (39.1%)
		Clinical Nurses	0 (0%)	10 (52.8%)	10 (43.5%)

### Availability LMIS tools (forms)

All assessed health facilities had manual LMIS forms like bin cards, stock cards, receiving and issuing vouchers (model 19 and model 22), report and resupply form (RRF), internal facility report and resupply forms (IFRRs). They were also using them. All assessed hospitals were using electronic-LMIS (computer) during the study period, while only 6 (31.58%) of HCs were using electronic-LMIS (Table 2).

**Table 2:** Availability and utilization of LMIS tools in public health facilities of West Wollega zone, Oromia regional state, Ethiopia, May, 2019

LMIS form	Available			Utilized		
	Hospitals	HCs	Total	Hospitals	HCs	Total
Bin cards	4 (100%)	19 (100%)	23 (100%)	4 (100%)	19 (100%)	23 (100%)
Stock card	4 (100%)	19 (100%)	23 (100%)	4 (100%)	19 (100%)	23 (100%)
e-Recording system	4 (100%)	8 (42.1%)	12 (52.17%)	4 (100%)	6 (31.58%)	10 (43.48%)
Issuing voucher	4 (100%)	19 (100%)	23 (100%)	4 (100%)	19 (100%)	23 (100%)
Receiving voucher	4 (100%)	19 (100%)	23 (100%)	4 (100%)	19 (100%)	23 (100%)
RRF	4 (100%)	19 (100%)	23 (100%)	4 (100%)	19 (100%)	23 (100%)
IFRR	4 (100%)	19 (100%)	23 (100%)	4 (100%)	19 (100%)	23 (100%)
IPLS SOP Manual	4 (100%)	19 (100%)	23 (100%)	4 (100%)	11 (57.89%)	15 (65.22%)

### Supervision

**Fig. 1:**
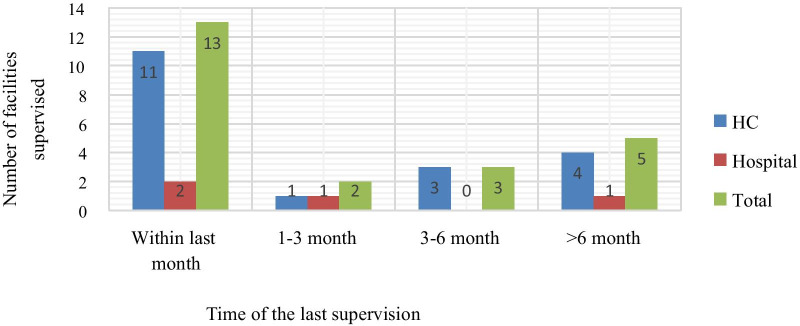
Time of last supervision for selected public health facilities of West Wollega zone, Oromia region, Ethiopia, May, 2019

Thirteen (56.52%) of the assessed facilities [two hospitals (50%) and eleven HCs (57.9%)] got supervision from different organizations within the last month; whereas, 5 (24.74%) were not supervised in the last 6 months from the study period (Fig. 1).

### Availability of FP/MCH medicines

On average FP/MCH medicines were available roughly in more than half 14 (61.30%) of facilities and ranges from 0 (0%) to 23 (100%). Four of assessed FP/MCH medicines; condom (female), ampicillin injection, metronidazole injection, and vitamin K injection were not available in all facilities, whereas condom (male), contraceptive pills, gentamycin injection, amoxicillin 250 mg dispersible tablet and zinc 20 mg scored dispersible tablet were available in all facilities (Table 3). Details of Table 3 are available in Additional file 1.

**Table 3:** Availability, stock status and bin card utilization for FP/MCH medicines at public health facilities of West Wollega zone, Oromia, Ethiopia, May 2019 (*N* = 23)

Name of medicine	*A*	*B*	*C*	*D*	*E*
Condom (female)	0 (0%)	23 (100%)	180	1.0	0 (0%)
Condoms (male)	23 (100%)	0 (0%)	.00	.00	23 (100%)
Etonogestrel—68 mg capsule	22 (95.7%)	6 (26.09%)	16.87	.30	22 (95.65%)
IUCD (CU380 A)	22 (95.7%)	4 (17.39%)	10.91	.26	19 (82.61%)
Levonorgestrel—75 mg/rod	18 (78.3%)	11 (47.82%)	57.35	.57	21 (91.30%)
Levonorgestrel ( d -norgestrel)0.3	18 (78.3%)	8 (34.78%)	34.70	.52	19 (82.61%)
Levonorgestrel (d-norgestrel)0.75	11 (47.8%)	17 (73.91%)	71.61	1.22	20 (86.96%)
Oral contraceptive pills	23 (100%)	3 (13.04%)	1.65	.17	23 (100%)
Medroxyprogesterone acetate	21 (91.3%)	10 (43.48%)	31.43	.52	22 (95.65)
Oxytocin 10 IU injection	15 (65.2%)	13 (56.52%)	45.78	.96	22 (95.65)
Ringer lactate IV	14 (60.9%)	15 (65.22%)	65.09	.83	22 (95.65)
Calcium gluconate injection	8 (34.8%)	16 (69.57%)	100.13	.87	18 (78.26%)
Magnesium sulfate	18 (78.3%)	12 (52.17%)	31.04	.52	22 (95.65)
Ampicillin 500 mg inj	0 (0%)	23 (100%)	180.00	1.00	4 (17.39%)
Gentamycin injection	23 (100%)	4 (17.39%)	8.09	.09	23 (100%)
Metronidazole injection	0 (0%)	23 (100%)	170.13	1.00	9 (39.13%)
Misoprostol 200 mcg tab	14 (60.9%)	19 (82.61%)	67.17	1.17	22 (95.65%)
UL cure kit	12 (52.2%)	19 (82.61%)	161.70	1.13	15 (65.22%)
Addis cure kit	11 (47.8%)	23 (100%)	129.52	1.09	18 (78.26%)
Addis cure plus kit	19 (82.6%)	17 (73.91%)	95.09	.91	19 (82.61%)
Benzathine benzyl penicillin	13 (56.5%)	13 (56.52%)	78.57	.57	18 (78.26%)
Dexamethasone inj	3 (13.0%)	23 (100%)	161.61	1.04	12 (52.17%)
Chlorhexidine 7.1% gel	19 (82.6%)	9 (39.13%)	18.13	.30	21 (91.30%)
Amoxicillin 250 mg DT*	23 (100%)	1 (4.35%)	2.13	.04	23 (100%)
Procaine benzyl penicillin	6 (26.1%)	20 (86.96%)	102.70	1.13	14 (60.87%)
ORS	21 (91.3%)	2 (8.7%)	2.70	.09	23 (100%)
Zinc: 20 mg scored DT*	23 (100%)	1 (4.35%)	.39	.04	23 (100%)
TTC eye ointment	13 (56.5%)	16 (69.57%)	70.09	.83	17 (73.91%)
Ceftriaxone 1 inj	6 (26.1%)	23 (100%)	110.17	1.91	16 (69.57%)
Vitamin A caps	18 (78.3%)	6 (26.09%)	25.04	.26	19 (82.61%)
Vitamin K inj	0 (0%)	23 (100%)	162.30	1.30	10 (43.48%)
Average	14.1 (61.30%)	13 (56.52%)	70.71	0.67	18.03 (78.40%)

*A*: availability of medicine on the day of visit; *B*: medicines stock-out in 6 months from the study period; *C*: duration of stock-out within 6 months; *D*: frequency of stock-out within 6 months; *E*: availability of Bin card for each product

*Dispersable tablet

### Stock-out rates

Within 6 months immediately before the study period, on average, FP/MCH medicines were out of stock in 13 (56.52%) facilities. One of FP/MCH medicine (male condom) was available in all facilities, whereas, seven of FP/MCH medicines were out of stock at least once in all facilities. They were out of stock for 70.71 days on average, with a range of 0 days for male condom to 180 days for female condom and Ampicillin injection. The most frequently stock-out item was Ceftriaxone injection with average frequency of 1.91 times (Table 3). The most reported reason for stock-out was a delay on the part of the main supplier to supply the SDP for all products. All hospitals reported that ampicillin injection, metronidazole injection and dexamethasone injection were stock-out because they were not available at source supplier.

### LMIS performance

#### Bin card utilization, updating practice and accuracy

**Fig. 2:**
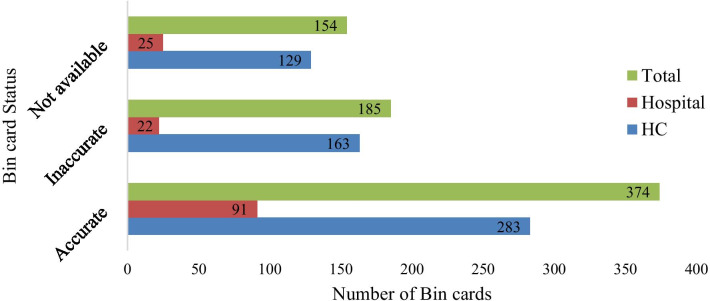
Bin card availability and accuracy for FP/MCH medicines at public health facilities of West Wollega zone, Oromia region, May, 2019

Five hundred fifty-nine bin cards were used for FP/MCH medicines in selected facilities from expected 713 bin cards. In other words, on average 18 (78.40%) facilities were using logistics recording tools (bin cards) (Table 3). Bin card accuracy was determined by calculating the discrepancy between physical stock count and ending balance recorded on bin card. Accordingly, 374 (52.45%) bin cards were accurate, 185 (25.95%) bin cards were inaccurate and 154 (21.60%) were not available (Fig. 2).

#### Reporting rate, completeness, timelines and accuracy

**Fig. 3:**
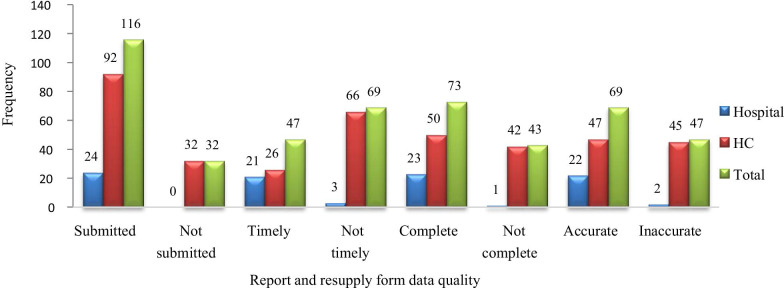
Report submission rate, timeliness, completeness, and accuracy for FP/MCH medicines at public health facilities of West Wollega zone, Oromia region, May, 2019

According to integrated pharmaceutical logistics system of Ethiopia, each facility should submit report and resupply forms every 2 months. So, 138 RRFs were expected from the assessed (23 facilities) in 1 year. But they reported only 116 RRFs, i.e., report submission rate of 84.06%. Among them only 47 (40.52%) RRFs were submitted on time and 73 (62.93%) were complete. LMIS reports (RRFs) were reviewed for accuracy of the logistics data by calculating the discrepancy between ending balance on bin card and stock on hand recorded on RRF. Then, 69 (59.48%) RRFs were found to be accurate and 47 (40.52%) RRFs were inaccurate (Fig. 3).

### FP/MCH inventory management challenges

Key informants were interviewed to explore the challenges they were facing regarding inventory management practices of FP/MCH medicines and the results were thematically categorized into the following themes.

### Supplier-related challenges

Most of the KIs complained that MCH program differs from other programs (like TB, HIV/AIDS, malaria) in supply of medicines. Even medicines in MCH program are not specific. The supply has more interruption and most of drugs are given from budget drugs, because the supplying agency does not supply them in program drugs. This can be exemplified by:

*“Supply of MCH drugs differs from other programs like TB, HIV/AIDS and Malaria medicines. Unlike that of these programs, the supply of MCH medicines is usually interrupted and PSA does not supply all products in quantity we requested. Also, most of the medicines are not supplied at all. For example, drugs like Ampicillin injection, Metronidazole injection, Benzathine Penicillin and Procaine penicillin are not being supplied through the program. Sometimes NGOs like Engender health brings some medicines. Just they bring what they have and they do not ask any report or request from us. I always report 0 balances, but they do not supply us. The drugs used in maternal health specially used during delivery are free of charge and it is challenging to buy trough RDF. PSA refills almost all FP medicines, but not MCH medicines.”*

Another KI from medical directors also stated that:

*“Currently most of the deliveries are at hospital. Because the service is free. But most of the drugs are given from RDF. By its nature RDF should rotate on itself, that means, the gov’t allocate budget once and we procure drugs with that budget, then sell the purchased drugs and purchase another batch. But due to poor supply of MCH drugs by program, we are left with the option of giving free of charge from RDF which is weakening the budget.”*

### Man power related

Another challenge raised by most of the KIs was human resource challenge. Most KIs complained shortage of professionals as a major contributing factor for poor inventory management. For example, one of the PHCU directors explained:

*“We have no pharmacy professional in our facility. We assigned clinical nurses both at dispensary and store in order to avoid service interruption. We have been asking ZHD to hire druggists. They haven’t hired for us. I think there is shortage of pharmacy professionals currently. Those assigned nurses are trying to do all duties of pharmacy, but they are facing technical difficulties. Whenever there is training on pharmacy issues, we send them and they are improving their capacity.”*

### Administrative related

In addition to shortage of professionals, KIs explained that available professionals were not giving services to their full potential because of administrative challenges. This challenge was explained by one of KIs as follows:

*“we are too busy, e.g. I’m store manager but now I’m dispensing because a person assigned here is day off (taking break after night duty). Always either of us is day-off. Since they fear that our duty hour increases, so that our over time payment will be high, they allow us to take a day break after night duty. In addition to work load I’m also discouraged to update bin cards appropriately while dispensing.”*

### Infrastructure related

Another issue raised by KIs was infrastructure related problem. Most of the KIs from the HCs complained that lack of computer infrastructure, as a challenge for LMIS management. For example, one of the store managers said:

*“Updating bin cards manually and copying them to RRF regularly is boring. If I had a computer once I updated the bin card, it will be easy to retrieve, and RRF will be automatically printed. Sometimes, I commit simple mathematical errors when I update bin cards. These errors might not occur if I had used computer”.*

## Discussion

Inventory management plays a vital role in improving availability of medicines, reducing the cost of inventories, and improving patient care quality. In many countries, inventory management in the government facilities supply system is poor [6, 12]. This study assessed availability, stockout rates, and logistics management information system for family planning, maternal and child health medicines and its challenges.

### Availability and stock-out

Ensuring availability of medicines is the ultimate goal of an inventory management system in public health facilities. Products must be on hand when clients need them [11, 18]. This study revealed that, on average FP/MCH medicines were available in 14 (61.30%) of facilities and ranges from 0 (0%) to 23 (100%). This finding is slightly greater than study done in Uganda which indicated that availability of sexual and reproductive health commodities in public sector was 41% [19] and study done in India, which showed, the availability of medicines were 45.2% and 51.1% in two districts [20]. The difference may be because; the previous study included more products and a greater number of facilities than the current study.

Again, in the current study male condoms, COC, gentamycin injection, amoxicillin 250 mg dispersible tablet, and zinc 20 mg scored dispersible tablets were available in all facilities. The result is similar to a study conducted in Nigeria, which reported, more than 90% of health facilities had male condoms and COC [21] and another study done in Ethiopia, Amhara region, where male condoms and COC were available in most HCs, and emergency contraceptives available at 50% of the HCs [22]. The result is also in line with a study done in Ethiopia, at the national level, that showed, availability of priority life-saving RMNCH medicines and commodities ranges from 0 to 100%. Opposite to the present finding, in the previous study, amoxicillin dispersible tablets, and chlorhexidine 7.1% gel were not available at all hospitals [7]. The difference could be because the previous study was conducted by PFSA and partners (primary suppliers in the country) so, they might have had responded to stock-out of those medicines.

The availability of FP medicines was 17.55 (76.3%). The result is different from a similar study done in Bangladesh, which showed that 100% of family planning commodities were available at all sites [23]. The difference might be due to the number and type of products included in the study and selection of the study area. In the previous study, the number of products included was limited and the study area was purposively selected, where there was project work that might have improved the supply of those products.

Availability of drugs used in STI prevention and treatment; Ulcure kit, Addis cure kit, Addis cure plus kit were 12 (52.2%), 11 (47.8%), 19 (82.6%) with stock-out rate within 6 months of 19 (82.61%), 23 (100%), 17 (73.91%), respectively. Similar findings were reported by the study done in Uganda, which revealed the availability of commodities to treat STIs ranged from 45 to 95% in the public health facilities [19].

Regarding the duration of stock-out, the current study showed FP/MCH medicines were out of stock for 70.71 days on average with a range of 0 days for the male condom to 180 days for female condoms and Ampicillin injection within the preceding 6 months from the study period. The current finding is similar to a study conducted at the national level which showed, average stock-out periods was ranging from 7 to 365 days for the preceding 1 year [7]. But it is by far greater than the findings of a study done in Uganda, which reported, the stock-out duration ranged from 7 to 20 days [24]. The difference might be due to the number of products included in the current study is more than that of the previous study. The current finding is also greater than a study conducted by Gurmu and Ibrahim in the east Shewa zone, in which the average stock-out duration was 35.31 days [25]. The difference could be because of the previous study was conducted on key essential medicines, which has greater attention than FP/MCH medicines and the current study accounted only those stocks in program drug. There was high mean duration of stock-out which indicates poor responsiveness of the supplier to stock-out, as most of the KIs are complaining, as well as the reported RRFs showed successive zero reports.

The most reported reason for stock-out was a delay on the part of the main supplier to supply the SDPs for all products. All hospitals reported that Ampicillin injection, Metronidazole injection and Dexamethasone injection were stock-out because they were not available at source supplier in program drugs. This is similar to the findings of a study conducted in Indonesia [26], in Sierra Leone [27] and in Ethiopia at the national level [28]. This challenge was also addressed by KIs and, many of the KIs complained, supply interruption and some medicines were not supplied under program drug at all. They also stated that, the supply of MCH medicines was different from other programs and they were giving free of charge, but from budget drug (RDF) which was weakening RDF.

### LMIS performance

Logistics management information system performance of the assessed facilities was measured by reviewing logistics information for timeliness, completeness, and accuracy. Accordingly, only 559 (78.40%) bin cards were available from expected 713 bin cards. Among available bin cards, 374 (52.45%) were accurate. This is better than a study conducted in Nigeria which reported less than 50% of facilities completing record correctly, and 50% of the stores maintaining accurate stock cards [21]. The difference may be due to a gap in the availability of forms in the former study. Keeping accurate and timely records of stocks warns the logistics personnel about the stock status of the facility and helps to take corrective measures. The challenges underlying poor record-keeping in the current study were revealed through the qualitative method. Most KIs were complaining workload due to fear of overload payment by government as a challenge.

Regarding the quality of logistics report, the findings of the current study showed a report submission rate of 116 (84.06%), RRF completeness of 73 (62.93%), RRFs timeliness of 47 (40.52%) and accuracy of 69 (59.48%). These results are better than the study conducted in Nigeria which revealed poor reporting with only completeness and accuracy of 25% [21] and study conducted in South Sudan, that states, only 27% of assessed facilities accurately filled LMIS forms [29]. The possible reason might be, in the previous studies, there were gaps in the availability of LMIS forms that might have reduced record-keeping practice. But current results are less than the study conducted by Tiye and Gudeta, in East Wollega (report submission rate was 97%, Complete RRF of 97.8%, 69.4% submitted on time and 64.6% of RRFs were filled accurately) [30]. The difference might be because there was a security problem and political instability in the current study area, which might have affected the reporting habit and transportation. Another possible reason might be, in the previous study MCH program was not included which contributed a lion’s share in RRF incompleteness in the current study. Poor LMIS data regarding completeness, timeliness, and accuracy might be due to a shortage of professionals, poor supervision, untrained and non-pharmacy professionals as KIs complained shortage of human power as a challenge. KIs complained that, due to lack of pharmacy professionals at their facilities, they assigned nurses for managing medicines.

Poor LMIS data quality leads to distortion of logistics information and ultimately results in poor supply decisions by the supplier. This can, in turn, cause a shortage of some products and overage of others and expiration. This poor performance might be due to poor supervision, because timely supervision is important for strengthening inventory management, encouraging professionals to do their routine activities strictly, by ensuring the professionals have knowledge, skills, and materials, identifying weaknesses, and established logistics guidelines and procedures are being followed [31]. Especially supervisory visit is important to examine and providing on-the-job training and constructive feedback if an employee’s skills need improvement on core supply functions of inventory management at health facility level [32]. In present study, 13 (56.52%) of the assessed facilities [two (50%) hospitals and 11 (57.9%)] HCs were supervised within the last month from the study period. This might have had an impact on LMIS performance.

## Limitations of the study

The upper stream of the supply chain (PSA) and the lower stream (health posts) were not included. The duration of stock-out was estimated by the store manager where there were no documents for tracing. This might cause recall bias. Since many facilities didn’t update bin cards regularly and calculated average month of stock, some indicators like: excess inventory, below emergency order point, and near emergency order point were not calculated.

## Conclusion

From the current finding, we concluded that the inventory management performance of the assessed facilities was poor with a long time stock-out of FP/MCH medicines, and poor LMIS performance. The associated challenges identified were: lack of training, insufficient supply of medicines from main supplier, insufficient human resource, administrative bureaucracy, and lack of IT-infrastructure. Therefore, PSA and partners should give appropriate training for professionals working as medical store managers, and supply FP/MCH medicines in adequate quantity. The concerned government bodies should allocate proper budget for available professionals and IT-infrastructures for health centers.

## Supplementary Information

**Additional file 1.** **Supplementary file 1:** Measurement of Variable, details of the results, and list of medicines included in the study.

## Data Availability

The data sets generated and/or analyzed during the present study are available from the corresponding author on reasonable request.

## Abbreviations

FEFO: First Expiry First Out
HIV/AIDS: Human immune virus/acquired immuno-deficiency syndrome
IFRR: Internal facility report and re-supply form
IUDs: Intra-uterine device
LIAT: Logistics indicator assessment tool
LSAT: Logistics system assessment tool
PPH: Postpartum hemorrhage
RRF: Report and re-supply form
STI: Sexually transmitted infection
USAID: United State Agency for International Development
COC: Combined oral contraceptive pills
FMOH: Federal Ministry of Health
FP/MCH: Family planning/maternal and child health
FWC-FWV: Family Welfare-Center-Family Welfare Volunteer
HCs: Health centers
HFs: Health facilities
LARC/PM: Long-acting reversible contraceptive or permanent method
MCWC: Mother and Child Welfare Centers
MDG: Millennium Development Goal
MgSO4: Magnesium sulfate
MRH: Maternal and Reproductive Health
PHCU: Primary Health Care Unit
PSA: Pharmaceutical Supply Agency
RHCS: Reproductive Health Commodity Security
RIFs: Requisition and issue forms
RMNCH: Reproductive, Maternal, Neonatal and Child Health
SDPs: Service delivery points
SRHC: Sexual and reproductive health commodity
