# Supplementary material for: Inventory management performance for family planning, maternal and child health medicines in public health facilities of West Wollega zone, Ethiopia
Source: J Pharm Policy Pract. 2021 Feb 15;14:20. doi: 10.1186/s40545-021-00304-z (PMC7883421; doi:10.1186/s40545-021-00304-z)
Supplement: Supplementary file 1 — Additional file 1. Supplementary file 1: Measurement of Variable, details of the results, and list of medicines included in the study. [file 40545_2021_304_MOESM1_ESM.docx]

**Inventory Management Performance for Family Planning, Maternal and Child Health Medicines in Public Health Facilities of West Wollega Zone, Ethiopia**

**Variables and measurement of variables**

***Dependent Variables***

**Inventory management performance**

In this research inventory management performance was considered as a cumulative product of product stock status and Logistics management information System (LMIS) performance. Each of them is also measured by different performance indicators which are listed below (references 26, 29, 30 from the main document).

- Product stock status
- Availability: Availability of the medicines was assessed during data collection by physically counting medicines in store. Then medicines with physical stock counts of not zero were considered as available. Then the percentage was calculated for each product using the following formula.

Percentage of facilities with usable product available in stock at the time of review

$$= \frac{\# of facilities with usable product available in stock for specific product}{total number of facilities assessed}\boldsymbol{*}100$$

- Stock out Rates: The stock status of medicines within 6 months immediately before the study period, September 1, 2018 to April 30, 2019, were reviewed from documents. Accordingly, stock out medicines, duration of stock out and frequency of stock out were assessed. The stock out rate was then calculated as:

$\frac{number of facilities that experienced a stockout of a specific product}{total number of facilities that are expected to offer that product}$*100

- LMIS Performance: LMIS performance is measured by accuracy, timeliness and completeness of logistics data.
- Accuracy of logistics data

Inventory Accuracy Rate (or Accuracy of stock Balance for Inventory Management):

$$=\frac{number of items where stock record count equals physical stock count}{total number of items counted}\times100$$

Percentage of Facilities that had Accurate LMIS Reports:

$$=\frac{Number of facilities with discrepancies between LMIS report SOH with stock record card*100}{Total number of facilities submitted LMIS reports}$$

- LMIS Report submission rate:

=$\frac{Total Number of LMIS reports received*100}{No. of health \mathrm{facilities} expected to report X Number of reports expected from each}$

- Timeliness of logistics report

= $\frac{Total Number of reports received on time*100}{Total Number of reports expected}$

- Completeness of logistics report: logistics data were considered complete if they had he three essential data items, i.e. stock on hand, consumption, and loss/adjustment.

***Independent Variable***

**Service and practice related factors**

- Availability and Utilization of recording forms

Available/ not available

Utilized/not utilized

- Type of recording system

Manual/Electronic

- trainings provided

Trained/Not trained

- time of supervision from higher level

Within last month/1-3months ago/before 3 month

- Availability of guidelines and SOP manuals

SOP Manual available/not available

**Employee related factors**

- educational qualification

Diploma/Degree and above

- year of experience of the personnel

less than 1 year/ 1-5 years/ greater than 5 years

- Profession of the personnel

Pharmacy/ Non pharmacy

**Capacity related factors**

- type of HF

Health center/Hospital

**Details of the results**

**Table 1**: Availability of FP/MCH medicines on the day of visit at public health facilities of West Wollega Zone, May, 2019

| **S. No** | **Name of product** | **Availability** | | |
| --- | --- | --- | --- | --- |
|  |  | Hospital (N=4) | HC (N=19) | Total (N=23) |
| 1 | Condom (Female) | 0((0%) | 0(0%) | 0(0%) |
| 2 | Condoms (male) | 4(100%) | 19(100%) | 23(100%). |
| 3 | Etonogestrel68mg capsule (Implanon) | 4(100%) | 18(94.7%) | 22(95.7%) |
| 4 | IUCD (CU380 A) | 4(100%) | 18(94.7%) | 22(95.7%) |
| 5 | Levonorgestrel - 75 mg/rod of 2rods | 3(75%) | 15(78.9%) | 18(78.3%) |
| 6 | Levonorgestrel (D-Norgestrel)0.03mg | 3(75%) | 15(78.9%) | 18(78.3%) |
| 7 | Levonorgestrel (D-Norgestrel) 0.75mg | 3(75%) | 8(42.1%) | 11(47.8%) |
| 8 | Oral contraceptive pill | 4(100%) | 19(100%) | 23(100%) |
| 9 | Medroxyprogesterone Acetate-Injection | 4(100%) | 17(89.5%) | 21(91.3%) |
|  | **Average for FP** | **3.22 (80.6%)** | **14.33 (75.4%)** | **17.55(76.3%)** |
| 10 | Oxytocin 10 IU in 1ml ampule | 3(75%) | 12(63.2%) | 15(65.2%) |
| 11 | Ringer lactate 0.9% isotonic solution | 3(75%) | 11(57.9%) | 14(60.9%) |
| 12 | Calcium gluconate injection 100mg/ml | 3(75%) | 5(26.3%) | 8(34.8%) |
| 13 | Magnesium sulfate | 4(100%) | 14(73.7%) | 18(78.3%) |
| 14 | Ampicillin500mg inj. | 0(0%) | 0(0%) | 0(0%) |
| 15 | Gentamycin 10mg, 40mg/ml in 2ml | 4(100%) | 19(100%) | 23(100%) |
| 16 | Metronidazole injection 500 mg | 0(0%) | 0(0%) | 0(0%) |
| 17 | Misoprostol 200mcg tab | 4(100%) | 10(52.6%) | 14(60.9%) |
| 18 | UL cure kit | 4(100%) | 8(42.1%) | 12(52.2%) |
| 19 | Addis cure kit | 3(75%) | 8(42.1%) | 11(47.8%) |
| 20 | Addis cure plus kit | 4(100%) | 15(78.9%) | 19(82.6%) |
| 21 | Benzathine benzyl penicillin | 1(25%) | 12(63.2%) | 13(56.5%) |
| 22 | Dexamethasone inj. | 0(0%) | 3(15.8%) | 3(13.0%) |
| 23 | Chlorhexidine 7.1% gel | 3(75%) | 16(84.2%) | 19(82.6%) |
| 24 | Amoxicillin 250mg DT* | 4(100%) | 19(100%) | 23(100%) |
| 25 | Procaine benzyl penicillin injection | 1(25%) | 5(26.3%) | 6(26.1%) |
| 26 | ORS | 4(100%) | 17(89.5%) | 21(91.3%) |
| 27 | Zinc: 20 mg scored DT* or Equivalent | 4(100%) | 19(100%) | 23(100%) |
| 28 | TTC eye ointment | 3(75%) | 10(52.6%) | 13(56.5%) |
| 29 | Ceftriaxone 1inj. | 4(100%) | 2(10.5%) | 6(26.1%) |
| 30 | Vitamin A Caps | 3(75%) | 15(78.9%) | 18(78.3%) |
| 31 | Vitamin K inj. | 0(0%) | 0(0%) | 0(0%) |
|  | Average for MCH | 2.68 (67.1%) | 10 (52.6%) | 12.68 (55.1%) |
|  | **Overall Average** | **2.84 (71.0%)** | **11.26 (59.25%)** | **14.1 (61.30%)** |

**Table 2:** Stock out rate of FP/MCH medicines in preceding six months from the study period in public health facilities of west Wollega zone, May 2019

| S. No | Name of product | **6-month stock out rate** | | |
| --- | --- | --- | --- | --- |
|  |  | Hospital(N=4) | HC (N=19) | Total (N=23) |
| 1 | Condom (Female) | 4(100%) | 19(100%) | 23(100%) |
| 2 | Condoms (male) | 0(0%) | 0(0%) | 0(0%) |
| 3 | Etonogestrel68mg capsule (Implanon) | 0(0%) | 6(31.6%) | 6(26.09%) |
| 4 | IUCD (CU380 A) | 1(25%) | 3(15.8%) | 4(17.39%) |
| 5 | Levonorgestrel - 75 mg/rod of 2rods | 1(25%) | 10(52.6%) | 11(47.82%) |
| 6 | Levonorgestrel (D-Norgestrel)0.03mg | 2(50%) | 6(31.6%) | 8(34.78%) |
| 7 | Levonorgestrel (D-Norgestrel) 0.75mg | 1(25%) | 16(84.2%) | 17(73.91%) |
| 8 | Oral contraceptive pills | 0(0%) | 3(15.8%) | 3(13.04%) |
| 9 | Medroxyprogesterone Acetate-Injection | 2(50%) | 8(42.1%) | 10(43.48%) |
|  | **Average for FP** | **1.22 (30.5%)** | **7.89 (41.52%)** | **9.11 (39.61%)** |
| 10 | Oxytocin 10 IU in 1ml ampule | 2(50%) | 11(57.9%) | 13(56.52%) |
| 11 | Ringer lactate 0.9% isotonic solution | 1(25%) | 14(73.7%) | 15(65.22%) |
| 12 | Calcium gluconate injection 100mg/ml | 1(25%) | 15(78.9%) | 16(69.57%) |
| 13 | Magnesium sulfate | 0(0%) | 12(63.2%) | 12(52.17%) |
| 14 | Ampicillin500mg inj. | 4(100%) | 19(100%) | 23(100%) |
| 15 | Gentamycin 10mg, 40mg/ml in 2ml | 0(0%) | 4(21.1%) | 4(17.39%) |
| 16 | Metronidazole injection 500 mg | 4(100%) | 19(100%) | 23(100%) |
| 17 | Misoprostol 200mcg tab | 3(75%) | 16(84.2%) | 19(82.61%) |
| 18 | UL cure kit | 0(0%) | 19(100%) | 19(82.61%) |
| 19 | Addis cure kit | 4(100%) | 19(100%) | 23(100%) |
| 20 | Addis cure plus kit | 4(100%) | 13(68.4%) | 17(73.91%) |
| 21 | Benzathine benzyl penicillin | 3(75%) | 10(52.6%) | 13(56.52%) |
| 22 | Dexamethasone inj. | 4(100%) | 19(100%) | 23(100%) |
| 23 | Chlorhexidine 7.1% gel | 2(50%) | 7(36.8%) | 9(39.13%) |
| 24 | Amoxicillin 250mg DT* | 1(25%) | 0(0%) | 1(4.35%) |
| 25 | Procaine benzyl penicillin injection | 4(100%) | 16(84.2%) | 20(86.96%) |
| 26 | ORS | 0(0%) | 2(10.5%) | 2(8.7%) |
| 27 | Zinc: 20 mg scored DT* or Equivalent | 1(25%) | 0(100%) | 1(4.35%) |
| 28 | TTC eye ointment | 4(100%) | 12(63.2%) | 16(69.57%) |
| 29 | Ceftriaxone 1inj. | 4(100%) | 19(100%) | 23(100%) |
| 30 | Vitamin A Caps | 1(25%) | 5(26.3%) | 6(26.09%) |
| 31 | Vitamin K inj. | 4(100%) | 19(100%) | 23(100%) |
|  | Average for MCH | 2.32 (57.95%) | 12.27 (64.59%) | 14.59 (69.04) |
|  | **Overall Average** | **2 (50%)** | **11 (57.95%)** | **13 (56.52%)** |

**Table 3**: Mean duration of stock outs and frequency of stock out for FP/MCH medicines in the past six months preceding study period in Public health facilities of West Wollega zone, Oromia Regional state, May, 2019

| S. No | **Name of the product** | **Mean Duration of stock out (days)** | | | **Mean Number of times product was stock out** | | |
| --- | --- | --- | --- | --- | --- | --- | --- |
|  |  | Hosp. (N=4) | HC (N=19) | Total (N=23) | Hospital(N=4) | HC (N=19) | Total (N=23) |
| 1 | Condom (female) | 180 | 180 | 180 |  | 1.0 | 1.0 |
| 2 | Condoms (male) | 0 | .00 | .00 | .00 | .00 | .00 |
| 3 | Etonogestrel 68mg capsule (Implanon) | 0 | 20.42 | 16.87 | .00 | .37 | .30 |
| 4 | Intrauterine Contraceptive Device (CU380 A) | 0 | 12.26 | 10.91 | .25 | .26 | .26 |
| 5 | Levonorgestrel - 75 mg/rod of 2rods (Jedalle) | 0 | 66.32 | 57.35 | .25 | .63 | .57 |
| 6 | Levonorgestrel (D-Norgestrel)0.03mg tablet | 0 | 28.42 | 34.70 | .50 | .53 | .52 |
| 7 | Levonorgestrel (D-Norgestrel) 0.75mg tablet | 0 | 81.63 | 71.61 | .25 | 1.42 | 1.22 |
| 8 | Oral contraceptive pills | 2.25 | 2.00 | 1.65 | .00 | .21 | .17 |
| 9 | Medroxyprogesterone Acetate-Injection | 4.5 | 34.16 | 31.43 | .50 | .53 | .52 |
|  | **Average for FP** | 20.75 | 47.25 | 44.95 | .19 | .55 | .51 |
| 10 | Oxytocin 10 IU in 1ml ampule | 12.25 | 47.05 | 45.78 | .50 | 1.05 | .96 |
| 11 | Ringer lactate 0.9% isotonic solution | 14.75 | 69.32 | 65.09 | .25 | .95 | .83 |
| 12 | Calcium gluconate injection 100mg/ml | 18.5 | 111.74 | 100.13 | .25 | 1.00 | .87 |
| 13 | Magnesium sulfate | 24 | 37.58 | 31.04 | .00 | .63 | .52 |
| 14 | Ampicillin500mg inj. | 39.75 | 180.00 | 180.00 | 1.00 | 1.00 | 1.00 |
| 15 | Gentamycin 10mg, 40mg/ml in 2ml | 42.75 | 9.79 | 8.09 | .00 | .11 | .09 |
| 16 | Metronidazole injection 500 mg | 45 | 170.53 | 170.13 | 1.25 | .95 | 1.00 |
| 17 | Misoprostol 200mcg tab | 45 | 66.79 | 67.17 | 1.25 | 1.16 | 1.17 |
| 18 | UL cure kit | 45 | 159.11 | 161.70 | 1.00 | 1.16 | 1.13 |
| 19 | Addis cure kit | 60.75 | 129.79 | 129.52 | 1.00 | 1.11 | 1.09 |
| 20 | Addis cure plus kit | 64.5 | 89.53 | 95.09 | 1.50 | .79 | .91 |
| 21 | Benzathine benzyl penicillin | 69 | 66.68 | 78.57 | .75 | .53 | .57 |
| 22 | Dexamethasone inj. | 79.25 | 157.74 | 161.61 | 1.00 | 1.05 | 1.04 |
| 23 | Chlorhexidine 7.1% gel | 121.5 | 12.95 | 18.13 | .50 | .26 | .30 |
| 24 | Amoxicillin 250mg DT* | 128.3 | .00 | 2.13 | .25 | .00 | .04 |
| 25 | Procaine benzyl penicillin injection | 131.5 | 94.21 | 102.70 | 1.25 | 1.11 | 1.13 |
| 26 | ORS | 135 | 3.26 | 2.70 | .00 | .11 | .09 |
| 27 | Zinc: 20 mg scored DT* or Equivalent | 143 | .00 | .39 | .25 | .00 | .04 |
| 28 | TTC eye ointment | 168.3 | 68.16 | 70.09 | 1.00 | .79 | .83 |
| 29 | Ceftriaxone 1inj. | 174 | 120.58 | 110.17 | 2.25 | 1.84 | 1.91 |
| 30 | Vitamin A Caps | 45 | 20.84 | 25.04 | .26 | .26 | .26 |
| 31 | Vitamin K inj. | 180 | 168.79 | 162.30 | 1.75 | 1.21 | 1.30 |
|  | Average for MCH | 81.23 | 81.11 | 81.25 | 0.78 | 0.78 | 0.78 |
|  | **Overall Average** | **63.67** | **71.28** | **70.71** | **0.63** | **0.70** | **0.67** |

**Table 4**: Reason for stock out of FP/MCH medicines at West Wollega Zone Public health facilities of West Wollega zone, Oromia Region, May, 2019

| **S. No** | **Name of product** | **Reason for stock out** | | | | | | | |
| --- | --- | --- | --- | --- | --- | --- | --- | --- | --- |
|  |  | Delay on part of supplier | | Delay of SDP to request | | Expired | | Not available at main supplier | |
|  |  | HC (%) | Ho (%) | HC (%) | Ho (%) | HC (%) | Hosp (%) | HC (%) | Hosp (%) |
| 1 | Condom(female) | 0(0) | 0(0) | 19(100) | 4(100) | 0(0) | 0(0) | 0(0) | 0(0) |
| 2 | Etonogestrel 68mg | 2(10.5) | 1(25) | 4(21.1) | 0(0) | 0(0) | 0(0) | 0(0) | 0(0) |
| 3 | IUCD | 3(15.8) | 1(25) | 0(0) | 0(0) | 0(0) | 0(0) | 0(0) | 0(0) |
| 4 | Levonorgestrel75mg/rod | 7(36.8) | 1()25 | 0(0) | 0(0) | 0(0) | 0(0) | 0(0) | 0(0) |
| 5 | Levonorgestrel D Norgestrel 0.03mg | 4(21.1) | 2(50) | 0(0) | 0(0) | 0(0) | 0(0) | 0(0) | 0(0) |
| 6 | Levonorgestrel_D_Norgestrel 0.75mg | 14(52.6) | 0(0) | 0(0) | 0(0) | 2(10.2) | 0(0) | 0(0) | 0(0) |
| 7 | Oral contraceptive pills | 3(15.8) | 0(0) | 0(0) | 0(0) | 0(0) | 0(0) | 0(0) | 0(0) |
| 8 | Medroxy Progesterone Acetate | 6(31.6) | 2(50) | 2(10.5) | 0(0) | 0(0) | 0(0) | 0(0) | 0(0) |
| 9 | Oxytocin 10IU in1mL Ampule | 10(52.6) | 2(50) | 0(0) | 0(0) | 1(5.3) | 0(0) | 0(0) | 0(0) |
| 10 | Ringer Lactate solution | 13(68.5) | 1(25) | 0(0) | 0(0) | 0(0) | 0(0) | 0(0) | 0(0) |
| 11 | Calcium Gluconate Injection | 8(41.1) | 1(25) | 0(0) | 0(0) | 0(0) | 0(0) | 0(0) | 0(0) |
| 12 | Magnesium Sulfate injection | 9(47.4) | 0(0) | 0(0) | 0(0) | 3(15.8) | 0(0) | 0(0) | 0(0) |
| 13 | Ampicillin injection | 6(31.6) | 0(0) | 0(0) | 0(0) | 0(0) | 0(0) | 13(68.4) | 4(100) |
| 14 | Gentamycin injection | 1(5.3) | 0(0) | 1(5.3) | 0(0) | 0(0) | 0(0) | 0(0) | 0(0) |
| 15 | Metronidazole injection | 7(36.8) | 0(0) | 0(0) | 0(0) | 0(0) | 0(0) | 7(36.8) | 4(100) |
| 16 | Misoprostol | 16(84.2) | 4(100) | 0(0) | 0(0) | 0(0) | 0(0) | 0(0) | 0(0) |
| 17 | Ulcure kit | 10(52.6) | 4(100) | 7(36.8) | 0(0) | 0(0) | 0(0) | 0(0) | 0(0) |
| 18 | Addis cure kit | 12(63.2) | 4(100) | 7(36.8) | 0(0) | 0(0) | 0(0) | 0(0%) | 0(0) |
| 19 | Addis cure plus kit | 7(36.8) | 3(75) | 6(31.6) | 0(0) | 0(0) | 0(0) | 0(0%) | 0(0) |
| 20 | Benzathine benzyl Penicillin | 7(36.6) | 3(75) | 0(0) | 0(0) | 1(5.3) | 0(0) | 0(0%) | 0(0) |
| 21 | Dexamethasone injection | 17(89.5) | 4(100) | 2(10.5) | 0(0) | 0(0) | 0(0) | 0(0%) | 0(0) |
| 22 | Chlorhexidine 7.1% gel | 4(21.1) | 2(50) | 2(10.5) | 0(0) | 1(5.3) | 0(0) | 0(0%) | 0(0) |
| 23 | Procaine Benzyl Penicillin injection | 10(52.6) | 4(100) | 0(0%) | 0(0) | 0(0%) | 0(0) | 6(31.6) | 0(0) |
| 24 | Oral Rehydration Salt | 2(10.5) | 0(0) | 0(0%) | 0(0) | 0(0%) | 0(0) | 0(0) | 0(0) |
| 25 | Zinc:20mg scored dispersible tablet | 0(0%) | 0(0) | 0(0%) | 0(0) | 0(0%) | 0(0) | 0(0) | 0(0) |
| 26 | Tetracycline eye ointment | 12(632) | 3(75) | 0(0%) | 0(0) | 1(5.3) | 0(0) | 0(0) | 1(25) |
| 27 | Ceftriaxone injection | 8(42.1) | 4(100) | 0(0%) | 0(0) | 0(0%) | 0(0) | 11(57.9) | 0(0) |
| 28 | Vitamin A capsule | 4(21.1) | 1(25) | 0(0%) | 0(0) | 0(0%) | 0(0) | 0(0) | 0(0) |
| 29 | Vitamin K injection | 17(89.5) | 3(75) | 0(0%) | 0(0) | 0(0%) | 1(25) | 2(10.5) | 0(0) |

**Table 5**: Bin card utilization and updating practice for FP/MCH medicines at public Health facilities of West Wollega zone, Oromia region, Ethiopia, May, 2019

| **S. No** | **Name of Medicine** | **Bin card utilized** | | | **Bin card updated** | | |
| --- | --- | --- | --- | --- | --- | --- | --- |
|  |  | Hospital  (N=4) | HC  (N=19) | Total  (N=23) | Hospital (N=4) | HCs  (N=19) | Total (N=23) |
| 1 | Condom(female) | 0(0%) | 0(0%) | 0(0%) | 0(0%) | 0(0%) | 0(0%) |
| 2 | Condoms (male) | 4(100%) | 19(100%) | 23(100%) | 4(100%) | 7(36.8%) | 11(47.83%) |
| 3 | Etonogestrel - 68mg Capsule | 4(100%) | 18(94.7%) | 22(95.65%) | 4(100%) | 13(36.8%) | 17(73.91%) |
| 4 | IUCD (CU380 A) | 3(75%) | 16(84.2%) | 19(82.61%) | 3(75%) | 11(57.9%) | 14(60.87%) |
| 5 | Levonorgestrel - 75 mg/rod | 3(75%) | 18(94.7%) | 21(91.30%) | 3(75%) | 14(73.7%) | 17(73.91%) |
| 6 | Levonorgestrel (DNorgestrel)0.3 | 3(75%) | 16(84.2%) | 19(82.61%) | 3(75%) | 10(52.6%) | 13(56.52%) |
| 7 | Levonorgestrel (DNorgestrel)0.75 | 4(100%) | 16(84.2%) | 20(86.96%) | 4(100%) | 11(57.9%) | 15(65.22%) |
| 8 | Oral contraceptive pills | 4(100%) | 19(100) | 23(100%) | 4(100%) | 12(63.2%) | 16(69.57%) |
| 9 | Medroxyprogesterone Acetate | 4(100%) | 18(94.7%) | 22(95.65) | 4(100%) | 12(63.2%) | 16(69.57%) |
| 10 | Oxytocin 10 IU injection | 4(100%) | 18(94.7%) | 22(95.65) | 4(100%) | 13(68.4%) | 17(73.91%) |
| 11 | Ringer lactate IV | 4(100%) | 18(94.7%) | 22(95.65) | 4(100%) | 13(68.4%) | 17(73.91%) |
| 12 | Calcium gluconate injection | 3(75%) | 15(78.9%) | 18(78.26%) | 3(75%) | 12(63.2%) | 15(65.22%) |
| 13 | Magnesium sulfate | 4(100%) | 18(94.7%) | 22(95.65) | 4(100%) | 12(63.2%) | 16(69.57%) |
| 14 | Ampicillin500mg inj. | 3(75%) | 1(5.3%) | 4(17.39%) | 3(75%) | 1(5.3%) | 4(17.39%) |
| 15 | Gentamycin injection | 4(100%) | 19(100%) | 23(100%) | 4(100%) | 16(84.2%) | 20(86.96%) |
| 16 | Metronidazole injection | 4(100%) | 5(26.3%) | 9(39.13%) | 4(100%) | 4(21.1%) | 8(34.78%) |
| 17 | Misoprostol 200mcg tab | 4(100%) | 18(94.7%) | 22(95.65) | 4(100%) | 12(63.2%) | 16(69.57%) |
| 18 | UL cure kit | 4(100%) | 11(57.9%) | 15(65.22%) | 4(100%) | 11(57.9%) | 15(65.22%) |
| 19 | Addis cure kit | 4(100%) | 14(73.7%) | 18(78.26%) | 4(100%) | 14(73.7%) | 18(78.26%) |
| 20 | Addis cure plus kit | 4(100%) | 15(78.9%) | 19(82.61%) | 4(100%) | 15(78.9%) | 19(82.61%) |
| 21 | Benzathine benzyl penicillin | 4(100%) | 14(73.7%) | 18(78.26%) | 4(100%) | 12(63.2%) | 16(69.57%) |
| 22 | Dexamethasone inj. | 3(75%) | 9(47.4%) | 12(52.17%) | 3(75%) | 9(47.4%) | 12(52.17%) |
| 23 | Chlorhexidine 7.1% gel | 3(75%) | 18(94.7%) | 21(91.30%) | 3(75%) | 13(68.4%) | 16(69.57%) |
| 24 | Amoxicillin 250mg DT* | 4(100%) | 19(100%) | 23(100%) | 4(100%) | 17(89.5%) | 21(91.30%) |
| 25 | Procaine benzyl penicillin | 4(100%) | 10(52.6%) | 14(60.87%) | 4(100%) | 10(52.6%) | 14(60.87%) |
| 26 | ORS | 4(100%) | 19(100%) | 23(100%) | 4(100%) | 13(68.4%) | 17(73.91%) |
| 27 | Zinc: 20 mg scored DT* | 4(100%) | 19(100%) | 23(100%) | 4(100%) | 15(78.9%) | 19(82.61%) |
| 28 | TTC eye ointment | 4(100%) | 13(68.4%) | 17(73.91%) | 4(100%) | 12(63.2%) | 16(69.57%) |
| 29 | Ceftriaxone 1inj. | 4(100%) | 12(63.2%) | 16(69.57%) | 4(100%) | 9(47.4%) | 13(56.52%) |
| 30 | Vitamin A Caps | 4(100%) | 15(78.9%) | 19(82.61%) | 4(100%) | 8(42.1%) | 12(52.17%) |
| 31 | Vitamin K inj. | 4(100%) | 6(31.6%) | 10(43.48%) | 4(100%) | 6(31.6%) | 10(43.48%) |
|  | **Average** | **3.65 (91.25%)** | **14.39 (75.74%)** | **18.03 (78.40%)** | **3.65 (91.25%)** | **10.55**  **(57.22%)** | **14.52**  **(63.11%)** |

**Table 6**: Bin card data accuracy for FP/MCH medicines at public Health facilities of West Wollega zone, Oromia region, Ethiopia, May, 2019

| Facility | Bin card  Accurate |  | Bin card inaccurate | Bin card not available |
| --- | --- | --- | --- | --- |
| Hospital 1 | 23(74.19%) |  | 5(16.13%) | 3(9.68%) |
| Hospital 2 | 27(87.10%) |  | 0(0%) | 4(12.90%) |
| Hospital 3 | 25(80.65%) |  | 1(3.23%) | 5(16.13%) |
| Hospital 4 | 18(58.06%) |  | 0(0%) | 13(41.93%) |
| HC 1 | 24(77.42%) |  | 2(6.45%) | 5(16.13%) |
| HC 2 | 18(58.06%) |  | 8(25.81) | 5(16.13%) |
| HC 3 | 11(35.48%) |  | 12(38.71%) | 8(25.81%) |
| HC 4 | 12(38.71%) |  | 14(45.16%) | 5(16.13%) |
| HC 5 | 23(74.19%) |  | 1(3.23) | 7(22.58%) |
| HC 6 | 10(32.26%) |  | 14(45.16%) | 7(22.58%) |
| HC 7 | 22(70.97%) |  | 5(16.13%) | 4(12.90%) |
| HC 8 | 7(22.58%) |  | 21(67.74%) | 3(9.68%) |
| HC 9 | 25(80.65%) |  | 2(6.45%) | 4(12.90%) |
| HC 10 | 22(70.97%) |  | 4(12.90%) | 5(16.13%) |
| HC 11 | 23(74.19%) |  | 5(16.13%) | 3(9.68%) |
| HC 12 | 13(41.94%) |  | 4(12.91%) | 14(45.16%) |
| HC 13 | 7(22.58%) |  | 19(61.29%) | 5(16.13%) |
| HC 14 | 5(16.13) |  | 19(61.29%) | 7(22.58%) |
| HC 15 | 16(51.61%) |  | 11(35.48%) | 4(12.90%) |
| HC 16 | 7(22.58%) |  | 3(9.68%) | 21(67.74%) |
| HC 17 | 22(70.97%) |  | 4(12.90%) | 5(16.13%) |
| HC 18 | 8(25.81%) |  | 17(54.84%) | 6(19.35%) |
| HC 19 | 6(19.35%) |  | 14(45.16%) | 11(35.48%) |
| Total | 374(52.45%) |  | 185(25.95%) | 154(21.60%) |

**List of Medicines Included in The Study**

**I.** Family planning:

1. Condoms (female),
2. Condoms (male),
3. Etonogestrel - 68mg - Capsule (Subdermal Implant),
4. IUCD (Intrauterine, Contraceptive Device) –
5. Long acting (CU380 A),
6. Levonorgestrel - 75 mg/rod of 2rods - implant rods (Sub dermal) with sterile insertion

trocar, Levonorgestrel (D-Norgestrel) - 0.03mg – Tablet,

1. Levonorgestrel (D-Norgestrel) - 0.75mg – Tablet,
2. Levonorgestrel(D-Norgestrel) +Ethinylestradiol +, Ferrous Fumerate - (0.15mg +

0.03mg +75mg) – Tablet (COC)

1. Medroxyprogesterone Acetate - 150mg/ml in 1ml Vial - Injection (Aqueous

suspension) with syringe (3 or2ml) 21G needle.

II. Maternal and Child Health medicines:

1. Oxytocin 10 IU in 1ml ampule
2. Sodium chloride 0.9% isotonic solution
3. Calcium gluconate injection 100mg/ml
4. Magnesium sulfate
5. Ampicillin 500mg injection.
6. Gentamycin 40mg/ml in 2ml
7. Metronidazole injection 500 mg in a 100-ml vial
8. Misoprostol 200mcg tablet
9. UL cure kit
10. Addis cure kit
11. Addis cure plus kit
12. Benzedrine benzyl penicillin
13. Dexamethasone injection
14. Chlorhexidine 7.1% gel
15. TTC eye Ointment
16. Amoxicillin 250mg dispersible tablet
17. Procaine benzyl penicillin: powder for injection 1 g and 3 g
18. Oral rehydration salt (ORS)
19. Zinc: 20 mg scored dispersible tablet or Equivalent
20. Ceftriaxone 1g injection.
21. Vitamin A Caps
22. Vitamin K injection
